# Different Types of Peptide Detected by Mass Spectrometry among Fresh Silk and Archaeological Silk Remains for Distinguishing Modern Contamination

**DOI:** 10.1371/journal.pone.0132827

**Published:** 2015-07-17

**Authors:** Li Li, Yuxuan Gong, Hao Yin, Decai Gong

**Affiliations:** 1 Basic Research Center of Heritage Conservation Science, Department for History of Science and Scientific Archaeology, University of Science and Technology of China, Hefei, China 230026; 2 Hefei National Laboratory for Physical Sciences at Microscale, University of Science and Technology of China, Hefei, China 230026; Shenzhen Institutes of Advanced Technology, CHINA

## Abstract

Archaeological silk provides abundant information for studying ancient technologies and cultures. However, due to the spontaneous degradation and the damages from burial conditions, most ancient silk fibers which suffered the damages for thousands of years were turned into invisible molecular residues. For the obtained rare samples, extra care needs to be taken to accurately identify the genuine archaeological silk remains from modern contaminations. Although mass spectrometry (MS) is a powerful tool for identifying and analyzing the ancient protein residues, the traditional approach could not directly determine the dating and contamination of each sample. In this paper, a series of samples with a broad range of ages were tested by MS to find an effective and innovative approach to determine whether modern contamination exists, in order to verify the authenticity and reliability of the ancient samples. The new findings highlighted that the detected peptide types of the fibroin light chain can indicate the degradation levels of silk samples and help to distinguish contamination from ancient silk remains.

## Introduction

Silk is one of the most famous biomaterial that widely used for thousands of years in human history. Raw silk consists of two types of self-assembled proteins: fibroin and sericin. Sericin contains a relatively large amount of hydrophilic amino acids and an unstable amorphous structure[1]. Fibroin consists of two subunits: a light chain (approximately 26 kDa) and a heavy chain (approximately 390 kDa). Twelve domains were identified in the heavy chain molecule that contains several Gly-X repeats, with X being Ala, Ser, Thr and Val[1–4]. These 12 domains that form the crystalline regions are linked with each other by the amorphous areas. The crystalline regions compose the β-sheet structure in which strong hydrogen bonds and Van der Waals forces generate a thermodynamically stable structure to exclude water, mild acidity or alkalinity and other degradation factors in the soil[1, 3, 5, 6]. The light chain and some regions in the heavy chain formed the amorphous regions of fibroin. Because the major components are polar amino acid residues, the amorphous regions are less organized in a looser structure and much easier to be damaged than crystalline regions[1, 3, 4, 7–9]. Furthermore, different from the amorphous regions in the heavy chain, the light chain is an independent sub-unit (only connected to the heavy chain by few disulfide bonds) and exhibited less stable properties, including more hydrophilic character, water uptake ability and degradation rate[4, 8, 10].

The amorphous regions of silk, especially the light chain, would degraded rapidly in the buried environments[7–9, 11]. Over long time degradation, crystalline regions would first be free crystal groups and progressively turned into invisible molecular residues[7, 8]. And the obtained rare residues (adsorbed by the soil, the bone, the copper artefacts and other materials[12, 13]) become the valuable evidence for revealing the ancient technologies and cultures. However, it is difficult to identify these invisible remains and exclude the modern contamination, which might be the fresh silk protein that sneaked into the ancient samples during the in-situ sample collection or the lab operation. Therefore, prior to any further analytical work, it is essential and significant to accurately identify the sample as genuine archaeological silk remains, rather than modern contaminations. Mass spectrometry (MS) is a powerful tool for the identification of ancient protein remains[13–19]. However, the identification approach could not directly determine the dating of the detected peptides and exclude the modern contamination which may affect the veracity of the identification. In this paper, a series of samples with a broad range of ages were carried out by MS to find an effective approach to determine whether modern contamination exists. The new findings highlighted that the number of residual peptide types of ancient silk remains were much lower than the fresh sample, especially for the extremely degraded silk. And the obvious changes of the light chain can help to distinguish contamination from ancient silk remains. Therefore, whether the sample is ancient silk remains or modern contamination (fresh silk protein) could be confirmed through this innovative approach on MS data without additional supplements. This would help to ensure the veracity of identifying the invisible silk remains in the archaeological samples.

## Materials and Methods

### Samples and chemicals

Three ancient samples were prepared: Yinan silk textile fragment (YN)(S1 Fig), Lu’an “huangwei” fragment (LA)(S2 Fig) and Hengshui pall imprint soil sample (HS)(S3 Fig). YN is a silk textile fragment from a dragon robe of Qing (1,636 AD–1,912 AD) unearthed in Yinan county, Linyi city, Shandong Province, China. LA is a fragment of the whole Huangwei (a type of pall made of silk fibers) unearthed from the M585 tomb of the “Warring States Period (403 BC-221 BC)” in Lu’an city, Anhui Province, China. The soil sample of Hengshui pall imprint (HS), a textile imprint on soil, was sampled from tomb M1, Peng-state Cemetery (1046 BC-771 BC), in Hengshui Town, Jiang County, Shanxi Province. Commercially available modern silk fibers from Guanghua Silk Co. Ltd., Hefei City, were used as fresh silk samples. All archaeological samples were collected during excavations, and they were fragment or soil samples that were used for experimental analysis. No permits were required for the described study. These three samples (YN, LA, HS) for experimental test were stored in the Basic Research Center of Heritage Conservation Science, Department for History of Science and Scientific Archaeology, University of Science and Technology of China, which is located in the No. 96 JinZhai Road Baohe District, Hefei, Anhui, P.R. China. These samples are accessible to other interested researchers.

Sodium carbonate (**Na**_**2**_ **CO**_**3**_),calcium chloride (**CaCl**_**2**_) forthe preparation of silk protein solution and ethanol (**EtOH**)were purchased from Sangon Biotech (Shanghai) Co. Ltd. Chymotrypsin was provided by Thermo Fisher Scientific. Calcium chloride (**CaCl**_**2**_) in chymotrypsin solution, **Tris**, **HCl**, formic acid (**FA**) and methyl alcohol(**MeOH**) were purchased from Sigma—Aldrich (St. Louis, MO).

### Methods

#### Sample preparation

For fresh silk YN and LA samples, 5 mg was degummed in 0.5% **Na**_**2**_ **CO**_**3**_ aqueous solution (liquor ratio 1:100) for 30 min and washed with water. The process was repeated once. Then, the samples were dried at room temperature.

For HS, 50 g soil sample was weighed and ground into powder.

#### Preparation of silk protein solution

To prevent fresh silk from contaminating ancient samples during experiments, all reused containers, including beakers and mortars were cleaned with a concentrated nitric acid/water solution (v/v = 1:1, heated to boiling).

The fresh silk, YN and LA samples were dissolved in 25 mL ternary solution (**CaCl**_**2**_:**H**_**2**_ **O**:**C**_**2**_ **H**_**5**_ **OH** molar ratio 1:8:2) at 95°C for 3 min[14, 20, 21]. The fibroin solutions were dialyzed with 14000 MWCO dialysis filters (Sangon Biotech, Shanghai) against 2000 mL deionized water for 48 h, and the deionized water was refreshed every 8 h. After dialysis, the precipitated material was removed by membrane separation with syringe filters (pore size of 0.45 μM). The fibroin solutions were concentrated to 100 μL with Amicon Ultra-15 centrifugal filters (Millipore, MWCO = 10 kDa) at 6000 rpm.

For the HS soil sample, the volume of the calcium/alcohol solution was increased to 50 mL, and the heating time was extended to 20 min[20, 21]. After centrifugation at 6000 rpm for 30 min, the supernatant was dialyzed with the same conditions mentioned above. Then, through a similar process, the fibroin solution was concentrated to 100 μL.

#### Digestion

All samples were treated using the same experimental procedure:

Fifty microliters concentrated solution of each sample was placed in a new Eppendorf tube and incubated with 1 μg chymotrypsin at 37°C for 20 h (digestion buffers: 10 mM calcium chloride and 500 m MTris•HCl, pH 8.0). The solution was then diluted with 0.1% formic acid for mass spectrometry. Here, it should be noted that the process of breaking the disulfide bonds followed by methylation does not affect the experimental results; thus, these steps were omitted[4].

#### NanoLC-MS/MS

All digested peptide mixtures were separated by online reversed-phase(RP) nanoscale capillary liquid chromatography(nanoLC) and analyzed by nano-electrospray ionization tandem mass spectrometry(NESI MS/MS). The samples were injected into a 10-cm reversed-phase, fused-silica capillary column (inner diameter 100 μm, packed in-house with a 5-μm Jupiter 300 Å C18,Phenomenex U.S.A.)using an Accela 600 pump (ThermoFisher Scientific, U.S.A.). The LC setup was connected to an LTQ-Qrbitrap XL mass spectrometer equipped with a nano-electrospray ion source(ThermoFisher Scientific). The peptides were separated with 155-min gradients from 10% to 90% B in 80 min. Solvent A was HPLC-grade H_2_O with 0.1% FA, and solvent B was LC-MS-grade MeOH. A 10-μL sample solution was loaded at a flow rate of 60 μL/min and eluted at a flow rate of 600nL/min. Data-dependent acquisition was performed on the LTQ-Orbitrap XL mass spectrometer in the positive ion mode. Survey MS scans were acquired in the Orbitrap with a resolution of 60,000. Each scan was recalibrated by an external standard. Up to the 5most intense ions per cycle were fragmented and analyzed in the linear ion trap. Target ions previously selected for MS/MS were dynamically excluded for 90 s. To reduce the effects of two different samples on each other, a blank sample was run after each sample[13–15, 22, 23].

#### Databasesearch and data analysis

Proteome Discoverer 1.2 (ThermoFisher Scientific) was used to extract peak lists from the LC-MS/MS data files for automated analysis. The SEQUEST algorithm was run on each of the datasets against the *B*. *mori*.fasta and the *fibroin*.fasta databases from the National Center for Biotechnology Information (*B*. *mori*.fasta: release date 01/10/2012; *fibroin*.fasta: release date 01/10/2012). For each run, no amino acid modifications were specified. Each peptide mass tolerance was < 3 ppm, and the fragment mass tolerance was < 0.8 Da. Except in rare instances, an accepted SEQUEST result was required to have a Δscore of ≥ 0.1[14, 15, 23].

The whole preparing process was shown in a flow chat (S4 Fig)

## Results

**Fig 1:**
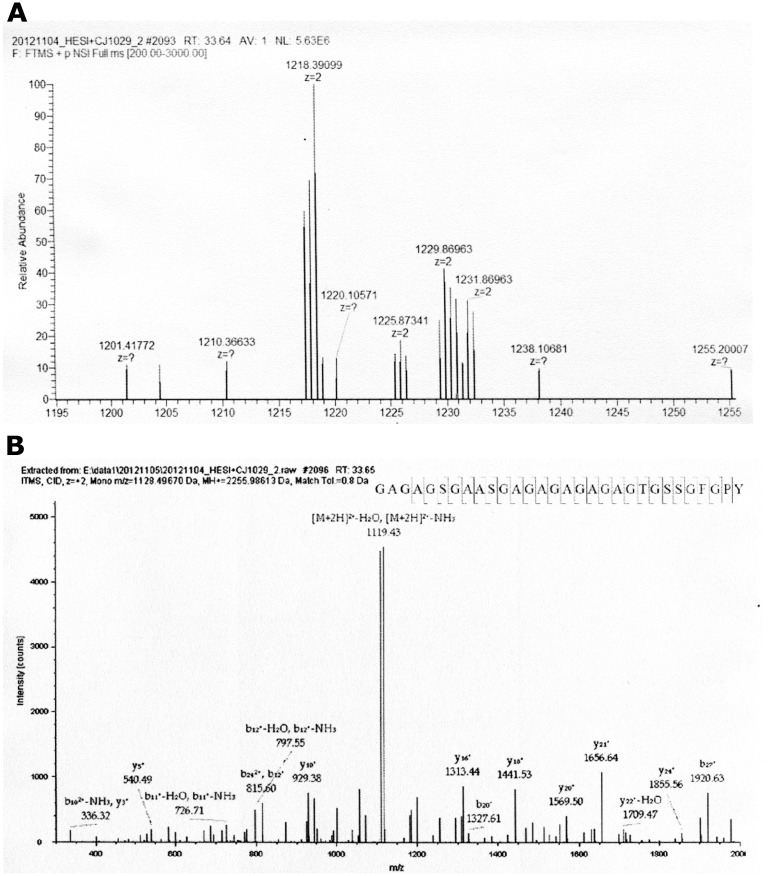
The Full MS and MS/MS results of Peptide GAGAGSGAASGAGAGAGAGAGTGSSGFGPY from fresh silk in Table 1. (A) The full MS data. (B) The b-type and y-type ion fragments of the peptide after CID in MS/MS.

Table 1 shows the detected peptides from each sample. Twenty-three unique silk fibroin peptides were detected in the fresh silk sample. Eleven peptides belong to the fibroin heavy chain, and the other 12 peptides belong to the fibroin light chain. Fig 1 shows the data-dependent results for the “GAGAGSGAASGAGAGAGAGAGTGSSGFGPY” peptide at m/z 1218.39099. As an example, Fig 1A shows the full MS data, and Fig 1B shows the b-type and y-type ion fragments of the peptide after CID in MS/MS. The deduced amino acid sequence and the breakpoints of the peptide are shown in Fig 1B.

**Table 1:** The detected peptide sequences of silk protein of fresh silk.

samples	Sequence	Protein Description
Fresh Silk	GAGAGSGAASGAGAGAGAGAGTGSSGFGPY	fibroin heavy chain precursor [Bombyxmori]
Fresh Silk	GAGAGSGAASGAGAGAGAGTGSSGFGPY	fibroin heavy chain precursor [Bombyxmori]
Fresh Silk	GQGAGSAASSVSSASSRSY	fibroin heavy chain precursor [Bombyxmori]
Fresh Silk	GIGVGAGYGAGAGVGY	fibroin heavy chain precursor [Bombyxmori]
Fresh Silk	MKTLSDGTVAQSY	fibroin heavy chain precursor [Bombyxmori]
Fresh Silk	GVGAGAGYGAGY	fibroin heavy chain precursor [Bombyxmori]
Fresh Silk	EYAWSSESDF	fibroin heavy chain precursor [Bombyxmori]
Fresh Silk	GAGAGAGY	fibroin heavy chain precursor [Bombyxmori]
Fresh Silk	GAGVGAGY	fibroin heavy chain precursor [Bombyxmori]
Fresh Silk	GAGVGAGYGAGAGSGAAF	fibroin heavy chain precursor [Bombyxmori]
Fresh Silk	VANGGYSRSDGY	fibroin heavy chain precursor [Bombyxmori]
Fresh Silk	NVQEILKDMASQGDY	fibroin light chain [Bombyxmori]
Fresh Silk	SDNEIPRDIDDGKASSVISRAW	fibroin light chain [Bombyxmori]
Fresh Silk	IAQAASQVHV	fibroin light chain [Bombyxmori]
Fresh Silk	DYVDDTDKSIAIL	fibroin light chain [Bombyxmori]
Fresh Silk	VINPGQLRY	fibroin light chain [Bombyxmori]
Fresh Silk	TDGVRSGNFAGF	fibroin light chain [Bombyxmori]
Fresh Silk	FGHVGQNL	fibroin light chain [Bombyxmori]
Fresh Silk	DFEAAW	fibroin light chain [Bombyxmori]
Fresh Silk	RQSLGPF	fibroin light chain [Bombyxmori]
Fresh Silk	RQSLGPFF	fibroin light chain [Bombyxmori]
Fresh Silk	VINPGQL	fibroin light chain [Bombyxmori]
Fresh Silk	HQSAGSITDLL	fibroin light chain [Bombyxmori]
YN	GAGAGSGAASGAGAGAGAGAGTGSSGFGPY	fibroin heavy chain precursor [Bombyxmori]
YN	GAGAGSGAASGAGAGAGAGAGTGSSGF	fibroin heavy chain precursor [Bombyxmori]
YN	GQGAGSAASSVSSASSRSY	fibroin heavy chain precursor [Bombyxmori]
YN	GAASGTGAGYGAGAGAGY	fibroin heavy chain precursor [Bombyxmori]
YN	GAGAGSGAGSGAGAGSGAGAGY	fibroin heavy chain precursor [Bombyxmori]
YN	VAADAGAYSQSGPY	fibroin heavy chain precursor [Bombyxmori]
YN	GAGAGAGYGAGAGAGY	fibroin heavy chain precursor [Bombyxmori]
YN	GAGYGAGVGAGY	fibroin heavy chain precursor [Bombyxmori]
YN	GAGVGAGYGVGY	fibroin heavy chain precursor [Bombyxmori]
YN	GAGAGAGY	fibroin heavy chain precursor [Bombyxmori]
YN	GAGVGAGY	fibroin heavy chain precursor [Bombyxmori]
YN	IAQAASQVHV	fibroin light chain [Bombyxmori]
YN	RQSLGPF	fibroin light chain [Bombyxmori]
YN	DYVDDTDKSIAIL	fibroin light chain [Bombyxmori]
YN	VINPGQL	fibroin light chain [Bombyxmori]
YN	NLINQL	fibroin light chain [Bombyxmori]
YN	DFEAAWDAIL	fibroin light chain [Bombyxmori]
YN	DFEAAW	fibroin light chain [Bombyxmori]
YN	NVQEIL	fibroin light chain [Bombyxmori]
LA	GIGVGAGYGAGAGVGY	fibroin heavy chain precursor [Bombyxmori]
LA	VITTDSDGNESIVEEDVLMKTL	fibroin heavy chain precursor [Bombyxmori]
LA	GAGAGSGAASGAGAGAGAGAGTGSSGFGPY	fibroin heavy chain precursor [Bombyxmori]
LA	EYAWSSESDF	fibroin heavy chain precursor [Bombyxmori]
LA	GAGAGAGY	fibroin heavy chain precursor [Bombyxmori]
LA	GAGVGAGY	fibroin heavy chain precursor [Bombyxmori]
HS	GAGAGSGAASGAGAGAGAGAGTGSSGF	fibroin heavy chain precursor [Bombyxmori]
HS	GAGAGSGAGSGAGAGSGAGAGY	fibroin heavy chain precursor [Bombyxmori]
HS	GAGAGAGY	fibroin heavy chain precursor [Bombyxmori]
HS	GAGVGAGY	fibroin heavy chain precursor [Bombyxmori]

All amino acid sequences are from NCBI (20121001). G, A, Y, S, V respectively are the abbreviations of glycine, alanine, tyrosine, serine and valine. The data details of each sample were shown in Supplementary Information (S1, S2, S3, S4 Tables).

Using the same MS/MS method, the identified peptides in ancient samples are showed in Table 1. The 11 detected peptides are all identified as silk fibroin heavy chain in sample YN. There were 7 detected peptide types of the fibroin light chain. Six and four peptides were detected from sample LA and HS, respectively, all of them belong to the fibroin heavy chain. The details of each samples were shown in Supplementary Information (S1, S2, S3, S4 Tables)

## Discussion

There is no damage observed in the fresh sample, and the intact fibroin provides the most abundant peptide information. As shown in Table 1, up to 23 peptides of fibroin were identified which were derived from the fibroin heavy chain and light chain, with no obvious correlation in specific areas of the whole fibroin amino acid sequence[2, 4]. For the ancient samples, with the breakdown of molecular chain and the loss of small compounds [7, 8, 11, 24], the detected peptide types decline gradually. And as mentioned above, the light chain has the less stable properties to make it more susceptible than the heavy chain and shows the most obvious changes of detected peptide types in the results.

YN sample was collected from the robe with an almost intact costume shape and relatively good mechanical strength (S1 Fig). The detected peptide types of the fibroin heavy chain in the YN sample exhibited no obvious changes, but slight damages still resulted in a decrease of the types of light chain peptides, which means the light chain was degraded before the heavy chain. Some visual degradation characteristics are present in the LA sample: dark brown color, poor strength and several falling powders (S2 Fig). The silk in HS soil sample was extreme degradation, and the macro-structure of the fibers were completely disappeared (S3 Fig). Limited peptide types were identified from the LA and HS samples. No peptides of the fibroin light chain were found and the identified peptides of the heavy chain were concentrated in the crystalline regions.

Exactly as the experimental results shown, in any case, the decline and disappearance of detected peptide types can be seen as the result of the degradation, and it is unlikely to occur when fresh silk proteins exist in the samples. To conclude, the detected peptide types of the light chain can serve as an effective method to verify the contamination: abundant light chain peptides were detected which means the ancient samples are likely to be contaminated by modern silk proteins.

**Fig 2:**
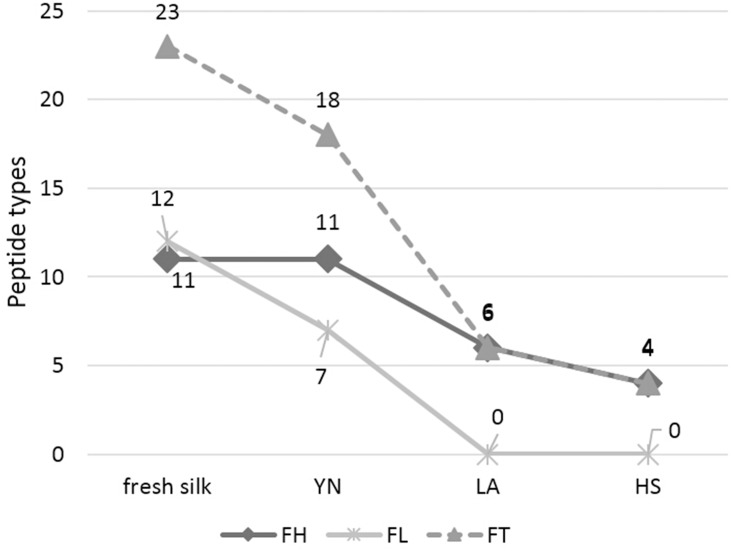
The tendency of detected amount of peptide types.

In addition, as the representative of different preservation conditions, the gradually decline of the peptide types detected in the three samples upon the aging intensifies, exhibited a clear downward trend as shown in Fig 2. The obtained preliminary findings also conform to fibroin’s degradation mechanisms. It may be the result of the breakdown of molecular chain which may reduce the compounds of fibroin and result in many property changes of silk fibroin, such as crystallinity, porosity and molecular weight[3, 7, 8]. Therefore, although this tendency is rough due to the limited sample size, it is reasonable and shows the changes of the detected peptide types.

## Conclusions

In summary, the fresh silk and three ancient silk samples with different durations of preservation were identified by mass spectrometry. The changes of detected peptide types showed a preliminary tendency of silk fibroin during the silk protein’s degradation process. Based on the existing degradation mechanisms, the changes of the detected peptide types, especially for the light chain, can indicate the fibroin degradation process and verify the existence of contamination when identify the ancient invisible silk remains. Without additional means, using the identification data is able to preliminarily determine whether the contamination exists. The achievement of this method will improve the reliability of identifying invisible silk remains from archaeological sites by MS and make it possible to explore the earlier silk evidence. It is worth mentioning that the finding in this study is preliminary and rough due to the rarity of ancient samples, but more samples will be analyzed in future works and more details of the changes of fibroin peptide types will be found and discussed clear.

## Supporting Information

S1 Fig The dragon robe of Qing (The sample YN). This robe has an almost intact costume shape and relatively good mechanical strength. And the sample we used is a fragment falling down from the robe.
(TIF)

S2 Fig The Lu’an Huangwei (The sample LA). Lu’an Huangwei is a type of wrought silk used to cover coffins. The experimental sample is a fragment around the whole textile. Some visual degradation characteristics are present in the LA sample: dark brown color, poor strength and several falling powders from textiles.
(TIF)

S3 Fig The soil sample of HengShui pall imprint (The sample HS). The fibroin degradation of the sample HS was much more serious. The macro-structure of the silk fiber had completely disappeared, and only the silk textile imprint was visible on the surface of the soil.
(TIF)

S4 Fig The flow chat of the preparing process. (TIF)

S1 Table The detected peptide sequences of silk protein of fresh silk. (PDF)

S2 Table The detected peptide sequences of silk protein of YN. (PDF)

S3 Table The detected peptide sequences of silk protein of LA. (PDF)

S4 Table The detected peptide sequences of silk protein in HS. (PDF)
