# Supplementary figures and images for: Different Types of Peptide Detected by Mass Spectrometry among Fresh Silk and Archaeological Silk Remains for Distinguishing Modern Contamination
Source: PLoS One. 2015 Jul 17;10(7):e0132827. doi: 10.1371/journal.pone.0132827 (PMC4505881; doi:10.1371/journal.pone.0132827)

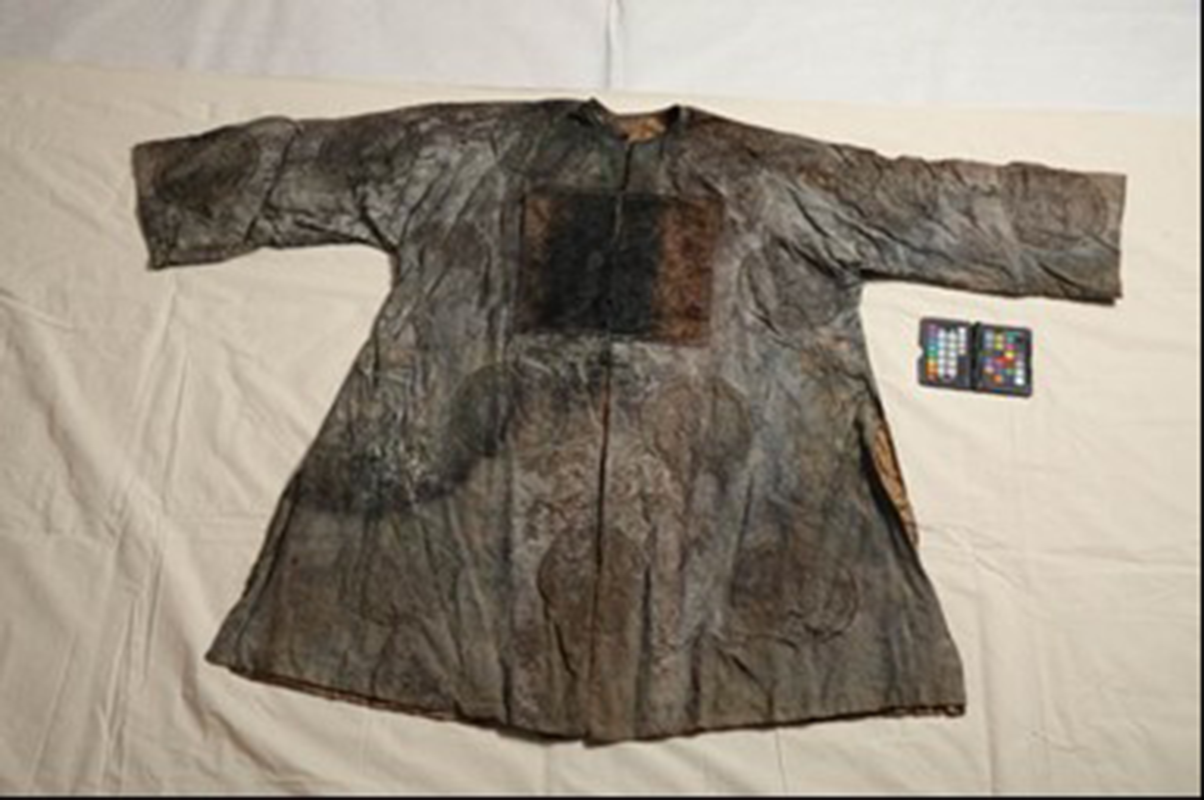

Supplement: S1 Fig — This robe has an almost intact costume shape and relatively good mechanical strength. And the sample we used is a fragment falling down from the robe. (TIF) [file pone.0132827.s001.tif]

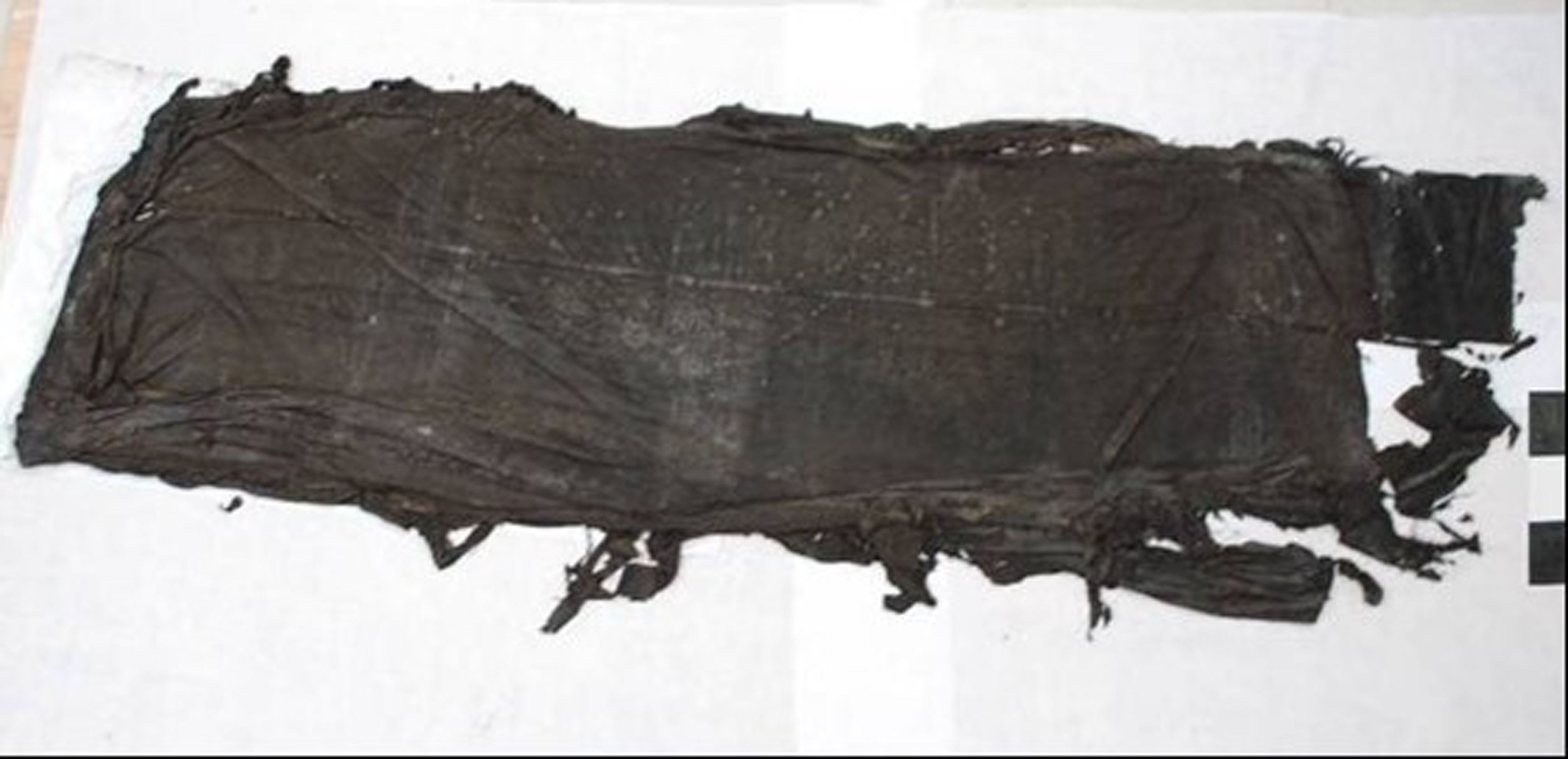

Supplement: S2 Fig — Lu’an Huangwei is a type of wrought silk used to cover coffins. The experimental sample is a fragment around the whole textile. Some visual degradation characteristics are present in the LA sample: dark brown color, poor strength and several falling powders from textiles. (TIF) [file pone.0132827.s002.tif]

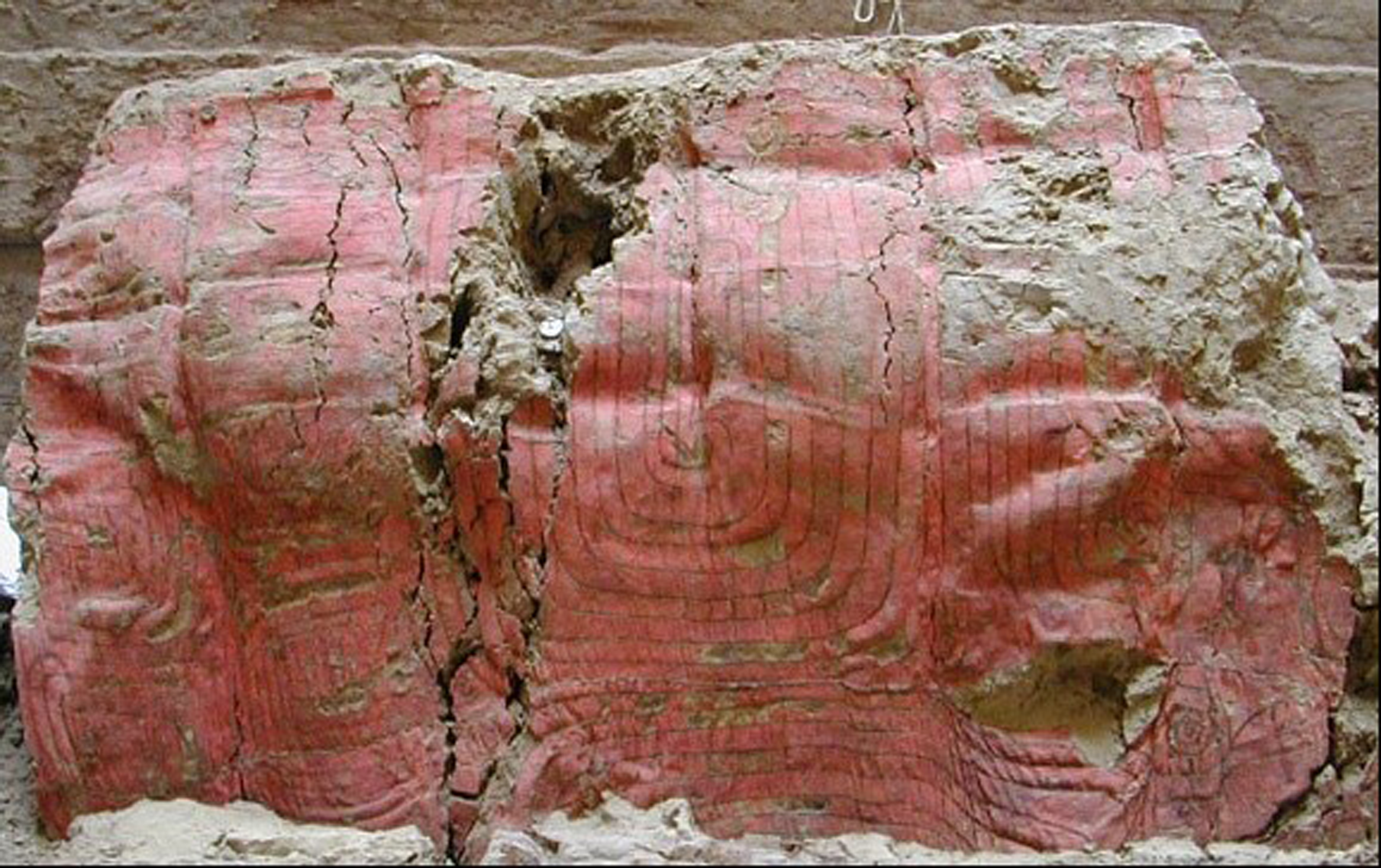

Supplement: S3 Fig — The fibroin degradation of the sample HS was much more serious. The macro-structure of the silk fiber had completely disappeared, and only the silk textile imprint was visible on the surface of the soil. (TIF) [file pone.0132827.s003.tif]

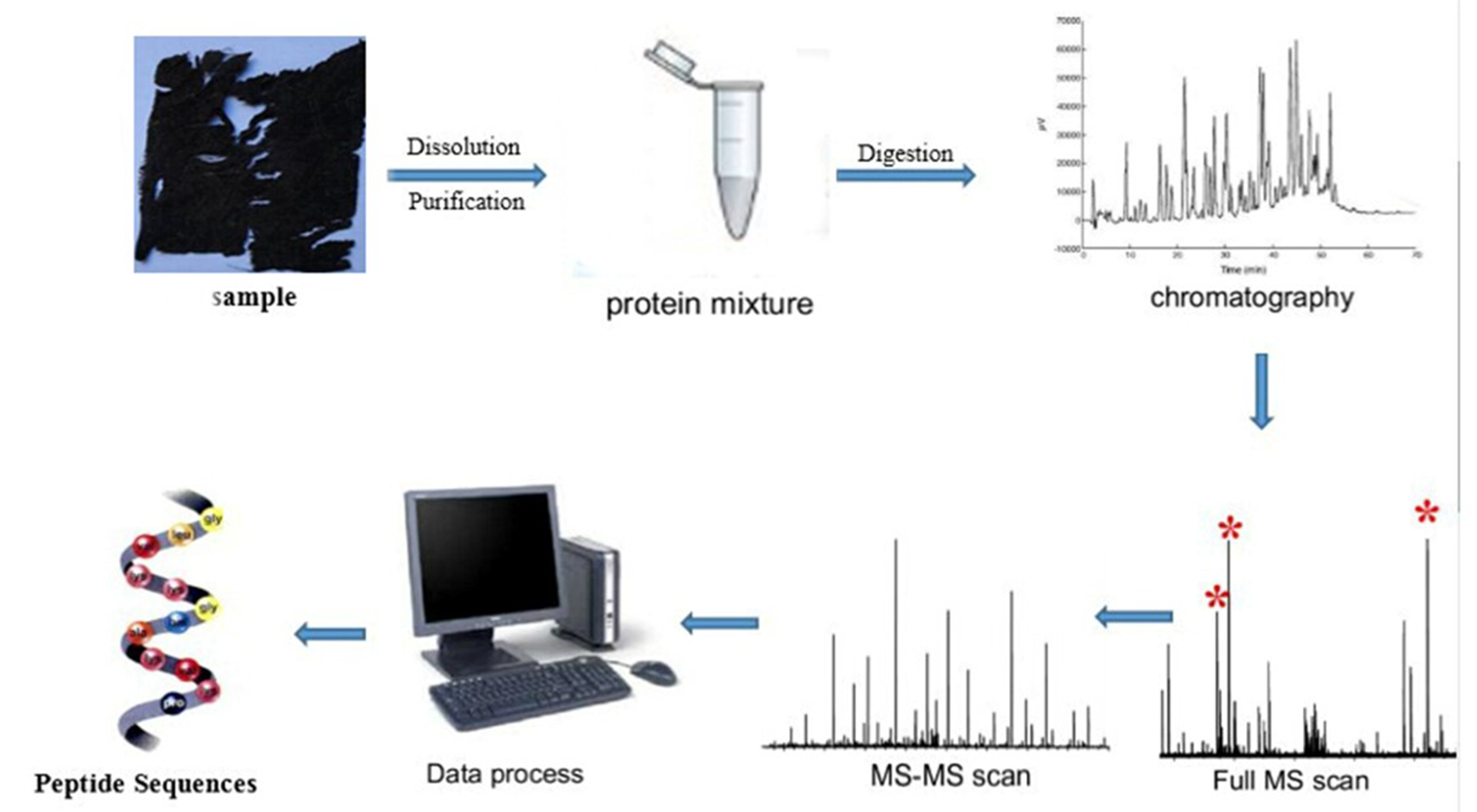

Supplement: S4 Fig — (TIF) [file pone.0132827.s004.tif]
