# Supplementary material for: Different Types of Peptide Detected by Mass Spectrometry among Fresh Silk and Archaeological Silk Remains for Distinguishing Modern Contamination
Source: PLoS One. 2015 Jul 17;10(7):e0132827. doi: 10.1371/journal.pone.0132827 (PMC4505881; doi:10.1371/journal.pone.0132827)
Supplement: S1 Table — (PDF) [file pone.0132827.s005.pdf]

S1 Table The detected peptide sequences of silk protein of fresh silk

Sequence	Protein Description	Protein Accessions	ΔScore	Charge	m/z [Da]	MH+ [Da]	ΔM [ppm]
GAGAGSGAASGAGAGAGAGAGTGSSGFGPY	fibroin heavy chain precursor [Bombyx mori]	gi164448672	1	2	1128.49670	2255.98613	-2.31
GAGAGSGAASGAGAGAGAGTGSSGFGPY	fibroin heavy chain precursor [Bombyx mori]	gi164448672	1	2	1064.46729	2127.92729	-2.56
GQGAGSAASSVSSASSRSY	fibroin heavy chain precursor [Bombyx mori]	gi164448672	1	2	858.89258	1716.77788	-0.31
GIGVGAGYGAGAGVGY	fibroin heavy chain precursor [Bombyx mori]	gi164448672	1	2	663.32599	1325.64470	-2.87
MKTLSDGTVAQSY	fibroin heavy chain precursor [Bombyx mori]	gi164448672	1	2	700.83984	1400.67241	-0.18
GVGAGAGYGAGY	fibroin heavy chain precursor [Bombyx mori]	gi164448672	0.1	2	500.22931	999.45134	-1.74
EYAWSSESDF	fibroin heavy chain precursor [Bombyx mori]	gi164448672	1	2	610.73907	1220.47087	-2.78
GAGAGAGY	fibroin heavy chain precursor [Bombyx mori]	gi164448672	0.33	1	623.27704	623.27704	-2.19
GAGVGAGY	fibroin heavy chain precursor [Bombyx mori]	gi164448672	0.17	1	651.30835	651.30835	-2.08
GAGVGAGYGAGAGSGAAF	fibroin heavy chain precursor [Bombyx mori]	gi164448672	1	2	699.32434	1397.64141	-2.22
VANGGYSRSDGY	fibroin heavy chain precursor [Bombyx mori]	gi164448672	1	2	623.27722	1245.54717	-1.87

NVQEILKDMASQGDY	fibroin light chain [Bombyx mori]	gi24637964	1	2	855.90198	171079668	-1.47
SDNEIPRDIDDGKASSVISRAW	fibroin light chain [Bombyx mori]	gi24637964	1	3	811.06549	2431.18192	-1.24
IAQAASQVHV	fibroin light chain [Bombyx mori]	gi24637964	1	2	512.28156	1023.55583	-2.32
DYVDDTDKSIAIL	fibroin light chain [Bombyx mori]	gi24637964	1	2	734.36285	1467.71843	-2.02
VINPGQLRY	fibroin light chain [Bombyx mori]	gi24637964	1	2	530.29956	1059.59184	-2.59
TDGVRSGNFAGF	fibroin light chain [Bombyx mori]	gi24637964	1	2	614.28967	1227.57207	-2.67
FGHVGQNL	fibroin light chain [Bombyx mori]	gi24637964	1	2	436.22403	871.44078	-1.52
DFEAAW	fibroin light chain [Bombyx mori]	gi24637964	1	1	738.30804	738.30804	-1.80
RQSLGPF	fibroin light chain [Bombyx mori]	gi24637964	1	2	402.72095	804.43462	-2.09
RQSLGPFF	fibroin light chain [Bombyx mori]	gi24637964	1	2	476.25504	951.50279	-2.03
VINPGQL	fibroin light chain [Bombyx mori]	gi24637964	1	1	740.42865	740.42865	-2.03
HQSAGSITDLL	fibroin light chain [Bombyx mori]	gi24637964	1	2	571.29456	1141.58183	-2.62
