# Supplementary material for: Different Types of Peptide Detected by Mass Spectrometry among Fresh Silk and Archaeological Silk Remains for Distinguishing Modern Contamination
Source: PLoS One. 2015 Jul 17;10(7):e0132827. doi: 10.1371/journal.pone.0132827 (PMC4505881; doi:10.1371/journal.pone.0132827)
Supplement: S2 Table — (PDF) [file pone.0132827.s006.pdf]

S2 Table The detected peptide sequences of silk protein of YN

Sequence	Protein Description	Protein Accessions	ΔScore	charge	m/z [Da]	MH+ [Da]	ΔM [ppm]
GAGAGSGAASGAGAGAGA GAGTGSSGFGPY	fibroin heavy chain precursor [Bombyx mori]	gi164448672	1.00	2	1128.49805	2255.98882	-1.12
GAGAGSGAASGAGAGAGA GAGTGSSGF	fibroin heavy chain precursor [Bombyx mori]	gi164448672	1.00	2	969.92847	1938.84966	-2.13
GQGAGSAASSVSSASSRSY	fibroin heavy chain precursor [Bombyx mori]	gi164448672	1.00	2	858.89233	1716.77739	-0.59
GAASGTGAGYGAGAGAGY	fibroin heavy chain precursor [Bombyx mori]	gi164448672	1.00	2	708.31250	1415.61772	-0.67
GAGAGSGAGSGAGAGSGA GAGY	fibroin heavy chain precursor [Bombyx mori]	gi164448672	1.00	2	784.33936	1567.67143	-1.17
VAADAGAYSQSGPY	fibroin heavy chain precursor [Bombyx mori]	gi164448672	1.00	2	678.80634	1356.60539	-0.95
GAGAGAGYGAGAGAGY	fibroin heavy chain precursor [Bombyx mori]	gi164448672	0.73	2	614.27203	1227.53679	-1.77
GAGYGAGVGAGY	fibroin heavy chain precursor [Bombyx mori]	gi164448672	0.10	2	500.22882	999.45036	-2.72
GAGVGAGYGVGY	fibroin heavy chain precursor [Bombyx mori]	gi164448672	0.42	2	514.24500	1027.48271	-1.62
GAGAGAGY	fibroin heavy chain precursor [Bombyx mori]	gi164448672	0.26	1	623.27979	623.27979	2.22
GAGVGAGY	fibroin heavy chain precursor [Bombyx mori]	gi164448672	0.22	1	651.30865	651.30865	-1.61

IAQAASQVHV	fibroin light chain [Bombyx mori]	gi24637964	1	2	512.28156	1023.55583	-2.32
RQSLGPF	fibroin light chain [Bombyx mori]	gi24637964	1	2	402.72095	80443462	-2.09
DYVDDTDKSIAIL	fibroin light chain [Bombyx mori]	gi24637964	1	2	734.36340	1467.71953	-1.27
VINPGQL	fibroin light chain [Bombyx mori]	gi24637964	1	1	740.42957	740.42957	-0.79
NLINQL	fibroin light chain [Bombyx mori]	gi24637964	1	1	714.41345	714.41345	-1.45
DFEAAWDAIL	fibroin light chain [Bombyx mori]	gi24637964	1	2	575.77307	1150.53887	-2.36
DFEAAW	fibroin light chain [Bombyx mori]	gi24637964	1	1	738.30823	738.30823	-1.55
NVQEIL	fibroin light chain [Bombyx mori]	gi24637964	1	2	358.20251	715.39775	-1.06
