# Supplementary material for: Different Types of Peptide Detected by Mass Spectrometry among Fresh Silk and Archaeological Silk Remains for Distinguishing Modern Contamination
Source: PLoS One. 2015 Jul 17;10(7):e0132827. doi: 10.1371/journal.pone.0132827 (PMC4505881; doi:10.1371/journal.pone.0132827)
Supplement: S3 Table — (PDF) [file pone.0132827.s007.pdf]

S3 Table The detected peptide sequences of silk protein of LA

Sequence	Protein Description	Protein Accessions	ΔScore	Charge	m/z [Da]	MH+ [Da]	ΔM [ppm]
GIGVGAGYGAGAGVGY	fibroin heavy chain precursor [Bombyx mori]	gi164448672	1	2	663.32629	1325.64531	-2.41
VITTDSDGNESIVEEDVLMKTL	fibroin heavy chain precursor [Bombyx mori]	gi164448672	0.85	2	1212.58545	2424.16362	-2.60
GAGAGSGAASGAGAGAGAGAGTGSSGFGPY	fibroin heavy chain precursor [Bombyx mori]	gi164448672	0.8	3	752.66638	2255.98459	-2.99
EYAWSSESDF	fibroin heavy chain precursor [Bombyx mori]	gi164448672	1	2	610.73944	1220.47161	-2.18
GAGAGAGY	fibroin heavy chain precursor [Bombyx mori]	gi164448672	0.25	1	623.27673	623.27673	-2.68
GAGVGAGY	fibroin heavy chain precursor [Bombyx mori]	gi164448672	0.15	1	651.30841	651.30841	-1.98
