# Supplementary material for: Different Types of Peptide Detected by Mass Spectrometry among Fresh Silk and Archaeological Silk Remains for Distinguishing Modern Contamination
Source: PLoS One. 2015 Jul 17;10(7):e0132827. doi: 10.1371/journal.pone.0132827 (PMC4505881; doi:10.1371/journal.pone.0132827)
Supplement: S4 Table — (PDF) [file pone.0132827.s008.pdf]

S4 Table The detected peptide sequences of silk protein in HS

Sequence	Protein Description	Protein Accessions	Δ Score	Charge	m/z [Da]	MH+ [Da]	ΔM [ppm]
GAGAGSGAASGAGAGA GAGAGTGSSGF	fibroin heavy chain precursor [Bombyx mori]	gi164448672	1	2	969.92999	1938.8527	-0.55
GAGAGSGAGSGAGAGS GAGAGY	fibroin heavy chain precursor [Bombyx mori]	gi164448672	1	2	784.34033	1567.6734	0.08
GAGAGAGY	fibroin heavy chain precursor [Bombyx mori]	gi164448672	0.21	1	623.27832	623.27832	-0.13
GAGVGAGY	fibroin heavy chain precursor [Bombyx mori]	gi164448672	0.15	1	651.30951	651.30951	-0.29
